# The Role of Prenatal Exposure to Lead and Manganese in Child Cognitive Neurodevelopment at 18 Months: The Results of the Italian PHIME Cohort

**DOI:** 10.3390/toxics13010054

**Published:** 2025-01-14

**Authors:** Valentina Rosolen, Fabiano Barbiero, Marika Mariuz, Maria Parpinel, Luca Ronfani, Liza Vecchi Brumatti, Maura Bin, Luigi Castriotta, Francesca Valent, D’Anna Latesha Little, Janja Snoj Tratnik, Darja Mazej, Ingrid Falnoga, Milena Horvat, Fabio Barbone

**Affiliations:** 1 Central Directorate for Health, Social Policies and Disability, Friuli Venezia Giulia Region, 34121 Trieste, Italy; valentina.rosolen@regione.fvg.it (V.R.); marika.mariuz@regione.fvg.it (M.M.); 2 UOC Unit of Occupational Medicine, Department of Medical Sciences, University of Trieste, 34127 Trieste, Italy; 3 Department of Medicine (DMED), University of Udine, 33100 Udine, Italy; maria.parpinel@uniud.it; 4 Institute for Maternal and Child Health, IRCCS ‘Burlo Garofolo’, 34127 Trieste, Italy; luca.ronfani@burlo.trieste.it (L.R.); liza.vecchibrumatti@burlo.trieste.it (L.V.B.); maura.bin@burlo.trieste.it (M.B.); 5 Institute of Hygiene and Evaluative Epidemiology, Friuli Centrale University Health Authority, 33100 Udine, Italy; luigi.castriotta@asufc.sanita.fvg.it; 6 Hygiene and Public Health, Friuli Centrale University Health Authority, 33100 Udine, Italy; francesca.valent@asufc.sanita.fvg.it; 7 Office of Chief Medical Officer, Western Friuli Health Authority, 33170 Pordenone, Italy; danna.little@asfo.sanita.fvg.it; 8 Department of Environmental Sciences, Jožef Stefan Institute, 1000 Ljubljana, Slovenia; janja.tratnik@ijs.si (J.S.T.); darja.mazej@ijs.si (D.M.); ingrid.falnoga@ijs.si (I.F.); milena.horvat@ijs.si (M.H.); 9 Jožef Stefan International Postgraduate School, 1000 Ljubljana, Slovenia; 10 Department of Medicine, Surgery and Health Sciences, University of Trieste, 34127 Trieste, Italy; fbarbone@units.it

**Keywords:** child neurodevelopment, Bayley scales of infant and toddler development, lead, manganese, trace element, cohort study

## Abstract

Prenatal lead (Pb) and manganese (Mn) exposure can impair neurodevelopment, targeting the central nervous system. This study investigated the effects of prenatal exposure to Pb and Mn on neurodevelopment in children at 18 months of age, using data from 607 Italian mother–child pairs enrolled in the Northern Adriatic Cohort II (NAC-II). All children born at term (≥37 weeks) were assessed with the Bayley Scales of Infant and Toddler Development, third edition. Cord blood concentrations of Mn and Pb were categorized as low or high exposures based on the 75th percentile of their distribution. Sociodemographic and lifestyle information was collected via questionnaires. Using simple and multiple linear regressions, the study examined the relationship between the cognitive composite score (COGN) and Mn and Pb co-exposure, including their interaction. Stratified regressions explored how Mn exposure influenced the effect of Pb, in the whole cohort and by the child’s sex. Beta coefficients (β) and the 90% confidence interval (90% CI) were estimated. Boys showed an interaction effect between Mn and Pb, with a reduction in COGN (β = −5.78, 90% CI: −11.17; −0.40), further described as a negative effect of high Pb on cognition when Mn exposure was also high (β = −6.98, 90% CI: −10.93; −3.04). No clear effects were observed in girls or the entire cohort at these levels of exposure. The findings highlight the harmful impact of combined prenatal Pb and Mn exposure on cognitive development in boys.

## 1. Introduction

The exposure of the general population to xenobiotic pollutants through the environment is a significant health concern. Air, food, and water are the main sources of exposure to most pollutants present in the living environment and prolonged exposure to these substances, even at low doses, can have effects on health [1]. Children are particularly susceptible to metal toxicity [2]; several studies have shown that, for children living in urban areas, air, dust, and atmospheric particulate matter constitute the main source of exposure to many metals with neurotoxic action, including Mn and Pb. The developing central nervous system (CNS) is the most vulnerable to damage from metal exposure [3]: accumulating evidence suggests that all age groups (independent of socioeconomic status, race, gender, and geographical location) and organs are susceptible to metal-induced toxicity, although the impact on the central nervous system is more severe and long-lasting in both the developing and adult human brain [4]. In pregnant and lactating woman, essential and toxic trace elements are transported to the placenta, and the mammary gland [5,6,7]. Among essential trace elements, Mn is a metal involved in human development, metabolism, and antioxidant mechanisms [8,9]. However, fetal exposure to excessive concentrations of Mn is neurotoxic and can cause cognitive and motor impairment [10,11]. Furthermore, excessive exposure to Mn in any phase of prenatal development may have detrimental effects on the neurodevelopment of children and may cause serious neurological disabilities [6,12,13]. On the other hand, some recent studies have reported a lack of an association between maternal prenatal levels of Mn and child neuropsychological development [14,15]. The biological mechanisms underlying fetal neurotoxicity due to Mn are not yet completely clear and the studies carried out have not led to unequivocal results, especially with regard to the possible effects of exposure to environmental concentrations, thus highlighting the need to further investigate this type of exposure [16].

Unlike Mn, Pb is not an essential micronutrient and exposure to this metal, first documented in the 1970s, is commonly recognized as neurotoxic [16,17]. To date, no level of exposure to Pb can be considered truly safe and free from neurotoxic effects, especially when it comes to childhood and adolescent exposure [18]. In children, Pb exposure has been associated with developmental delays, impaired cognitive function, attention deficit, hyperactive behavior, hearing impairment, and short stature [19,20,21,22]. The detrimental effect of prenatal low-exposure concentration to Pb on child neurodevelopment has also been shown [20,23,24,25]; therefore, any reduction in prenatal and early-life exposure to Pb is important to improve neurodevelopment in children [26]. Some findings suggest that prenatal Pb exposure and its interaction with genetic factors might jointly contribute to cognitive developmental delay (CDD) risk [27]. Furthermore, some studies suggest that the concurrent exposure to Mn and Pb can lead to severe impairments in cognitive function, behavioral problems, and motor skills [7], although this evidence in humans is limited [5]. The aim of the present research is to evaluate the association between child cognitive neurodevelopment at 18 months of age and the prenatal co-exposure to Mn and Pb in the Italian mother–child pairs enrolled in the Northern Adriatic Cohort II (NAC-II). The Italian NAC-II is part of the Mediterranean cohort involved in the “Public health impact of long-term, low-level, mixed element exposure in susceptible population strata” project (PHIME) [28], the goals of which were to investigate the extent of exposure to toxic metals and its impact of public health with particular focus on vulnerable groups.

## 2. Materials and Methods

### 2.1. Study Population

A detailed description of the study protocol and inclusion and exclusion criteria, has been published elsewhere [28,29]. Briefly, recruitment and follow-up took place at the Institute for Maternal and Child Health IRCCS Burlo Garofolo. At recruitment, eligible pregnant women, between 20 and 22 gestational weeks, filled in a short questionnaire to identify any excluding conditions and to provide some brief information on their families. At 20 to 32 gestational weeks, the pregnant women enrolled in the study were tested with the Standard Progressive Matrices (SPMs), a version of the Raven’s Progressive Matrices, to estimate the maternal intelligence quotient [30]. A cord blood sample was collected a few minutes after delivery and before clamping the umbilical cord.

At 1 month after delivery, the mothers filled in a detailed questionnaire to collect information on their demographic, socioeconomic, and health status, on pregnancy and delivery, and on their lifestyles, and dietary habits.

At 18 months after delivery, the mothers completed a supplementary questionnaire to detect changes in their sociodemographic information and socioeconomic status and the children were tested using the Bayley Scales of Infant and Toddler Development, third edition (BSID-III) [31].

Starting from 18 months after delivery, the home environment evaluation was performed using the AIRE instrument [32], an adaptation of the Home Observation for Measurement of the Environment (HOME) model [33]. The AIRE visit at home lasted 30 to 40 min. The AIRE, SPM, and BSID-III tests were conducted by trained psychologists.

### 2.2. Power Calculation

The NAC-II is, as already mentioned, part of the Mediterranean cohort involved in the PHIME project, the goals of which were to investigate the extent of exposure to toxic metals and its impact of public health. We estimated the sample size required to obtain a statistically significant result in the case of a moderate association with a relatively low level of exposure to mercury (THg). Assuming that the prevalence of a measurable neurodevelopmental delay is 10% among children whose mother’s hair has a THg level of ≥4.0 µg/g (n1) and 5% among children whose mother’s hair has a THg level of <4.0 µg/g (n2), and that the n2/n1 ratio = 1, α = 0.05, and β = 0.10, the sample size needed to estimate a risk ratio of 2.0 is n1 = 621, n2 = 621. Given the personnel and time available, we estimated we would be able to enroll approximately 1700 mother–child pairs in the whole Mediterranean cohort during the study period (750 from Italy (NAC-II), 350 from Slovenia, 200 from Croatia, and 400 from Greece) [28].

### 2.3. Ethics

The study protocol was approved by the Ethics Committees of the University of Udine and the Institute for Maternal and Child Health IRCCS Burlo Garofolo, Trieste (CE/V-70-05/02/2007).

Informed consent was obtained from all participants’ caregivers involved in the study. During the follow-up, participants and their parents were free to withdraw from participating in the study at any time upon request. The study was conducted according to the Declaration of Helsinki.

### 2.4. Exposures

In the present study, the exposures of interest are the levels of Mn and Pb in cord whole blood, measured in ng/g. The conversion of ng/g into µg/dl (both units of measurement are used in several studies in the literature), considering the blood density of 1.06, is given by the formula:

$$\mu g/\mathrm{dL}=\frac{\mathrm{ng}/g\times 0.1}{1.06}$$

### 2.5. Chemical Analysis

Cord blood samples were collected in Vacutainer Blue Cup NaH tubes, specific for metals determination and divided, after processing, into three aliquots of 1500 µL.

The aliquots of cord blood were stored in freezers (temperature −80 °C) and then transported on dry ice to the Jozef Stefan Institute (JSI) in Ljubljana for trace element determination. The determination of As, Cd, Cu, Mn, Pb, Se, and Zn in the cord blood samples was conducted by inductively coupled plasma–mass spectroscopy (ICP-MS). An aliquot of 0.3 mL of blood sample was diluted 10 times with an alkaline solution containing Triton X-100 and ethylenediaminetetraacetic acid disodium salt dehydrate (EDTA). An internal standard solution containing Ga, Gd, Y, and Sc was added. For calibration, the standard addition procedure was performed. Measurements of the prepared solutions were performed with an Octapole Reaction 5 System (ORS) inductively coupled plasma–mass spectrometer (7500ce, Agilent; Santa Clara, CA, USA) equipped with an ASX-510 Autosampler (Cetac; Omaha, NE, USA) (instrumental conditions: Babington nebulizer, Scott-type spray chamber, spray chamber temperature 5 °C, plasma gas flow rate 15 L/min, carrier gas flow rate 0.8 L/min, make-up gas flow rate 0.1 L/min, sample solution uptake flow rate 1 mL/min, RF power 1500 W, reaction cell gas helium 4 mL/min, isotopes monitored 55Mn, 63Cu, 66Zn, 75As, 77Se, 78Se, 111Cd, 114Cd, 206Pb, 207Pb, 208Pb). The device was calibrated daily using a solution containing Li, Mg, Y, Ce, Tl, and Co. The quantification of all isotopes was performed using 1 central point of the spectral peaks and 3 repetitions. The reference material Seronorm Trace Elements Whole Blood L-1 (Sero) was used to check the accuracy of the results, and the resulting values were in good agreement with the reference values. The LODs for Cd, Pb, As, Se, Cu, Zn, and Mn (calculated as 3 times the SD of the blank sample) were 0.12, 1.3, 0.13, 5, 11, 20, and 1.7 ng/g, respectively, in the blood samples [29].

### 2.6. Outcome Definition and Measurement

Children at 18 ± 2 months of age underwent the BSID-III neurodevelopment test, performed by 2 trained psychologists. Although cognitive, language, motor, social-emotional, and adaptive behavior composite scores were calculated, here, we report results only of the cognitive composite score as the study outcome. Composite scores have an average of 100, a standard deviation of 15, and a range from 40 to 160. The higher the composite score, the better the child’s neurodevelopment performance.

### 2.7. Covariates

The following list of covariates was considered for estimating the effect of Mn, Pb, and Mn–Pb interaction, measured in cord blood, on child neurodevelopment. Before pregnancy: the mother’s BMI. During pregnancy: the smoking habits (never, ex-smoker, and current smoker) and the smoking passive exposure at home and at work (Yes/No). At delivery: the mother’s age, mother’s educational title, size of the home as a socioeconomic factor, if the home was close (less than 1 km) to a source of pollution (yes/no), the area where the home was located (city center, suburbs, or rural area), and the child’s birth weight (g) and length (cm). At 18 months of age of the child: the number of months of breastfeeding (until 18 months of age) and who was mainly in charge of weekday childcare at 18 months (family member/caregiver or daycare). In addition, the IQ and AIRE scores were considered. The IQ score ranges between 62 and 128, with a mean of 100 (SD = 15) in the normative sample. The range of AIRE scores is 0–20 for each of the following subscales: communication and affective interaction between parents and child; the promotion of child autonomy when alone; respect for the child and the implementation of rules; the emotional atmosphere. The higher the scores, the better the family environment assessment.

### 2.8. Statistical Analysis

Consistently with a previous analysis performed in the Italian and Mediterranean cohort [29,34,35], only children born during or after week 37 of gestation who had at least one measure of Mn and Pb concentration in cord blood and underwent the BSID-III at 18 ± 2 months were included in the analysis of the present research.

The main characteristics of mother–child pairs were described by mean, standard deviation (SD), and median in the case of continuous variables and by frequency and percentage in the case of categorical variables. For Mn and Pb concentrations in cord blood, the 25th and 75th percentiles were also estimated. Since there are no reference values for prenatal exposure to Mn and Pb in umbilical cord blood, exposures to each metal were grouped into two classes: (i) below the 75th percentile and (ii) above and equal to the 75th percentile of the distributions of the concentrations of each metal in the whole cohort, representing the low and high exposure groups of children, respectively [36]. The mean, SD, and median of the cognitive composite scores in the groups of children with low and high exposures to Mn and Pb were also estimated.

The main characteristics of mother–child pairs were provided both for the whole cohort and by the child’s sex.

Simple and multiple linear regressions were performed to study the relationship between the cognitive composite score and the categorized Mn and Pb co-exposure in cord blood. The interaction between categorized Mn and Pb exposures was also included in the linear regression models. In the multiple regression model, VanderWeele’s automatic selection method [37] was applied and only covariates with a *p*-value < 0.15 were included in the final model. The categorized Mn and Pb exposures and their interaction were forced to remain in the final model. Beta coefficients and the 90% confidence interval (90% CI) were estimated. To investigate the effect of Mn exposure on the cognitive composite score depending on each level of Pb exposure, stratified multiple linear regressions for Pb levels were conducted. Stratified multiple linear regressions for Mn levels were also performed to investigate the effect of Pb exposure depending on each level of Mn exposure. Separate simple and multiple linear regressions were also applied for the cognitive composite score and each metal’s exposure. Beta coefficients and the 90% confidence interval (90% CI) were estimated. The *p*-value considered to indicate statistical significance was 0.10.

Finally, in order to investigate if the effect of Mn and Pb was different between males and females, stratified analyses by the child’s sex were performed.

Statistical analyses were carried out using SAS software version 9.4 (SAS Institute Inc., Cary, NC, USA).

## 3. Results

In the recruiting period, 900 pregnant women were enrolled into the cohort; 767 (85%) of these remained in the study at delivery, and 632 children, 82% of those born within cohort, underwent BSID-III testing at 18 months. The mothers of children lost to follow-up had significantly lower IQs (median 118 vs. 125, *p* = 0.0015) and were less frequently married (85.5% vs. 90.4%) and more frequently separated (8.4% vs. 3.3%, *p* = 0.0371) than the mothers of children still within the study at the age of 18 months. Age at delivery and employment status in the two groups of mothers, on the other hand, did not differ significantly. Mn concentrations in cord blood did not differ significantly in the two groups of mothers, while mothers lost to follow-up had significantly lower Pb concentrations (median 9.01 ng/g vs. 10.50 ng/g, *p* = 0.0088) than those still within the study.

After excluding preterm births, 460 children who had at least one measure of Mn and Pb concentration in cord blood and had undergone the BSID-III at 18 ± 2 months were used in the final analysis. The main characteristics of the 460 mothers included in the present research are shown for the whole cohort and by child’s sex in Table 1. The main characteristics of the 460 children considered in the final analysis in the whole cohort and by the child’s sex are shown in Table 2.

The distribution of cognitive composite scores and metal concentrations measured in cord blood are shown in Table 3. The concentrations are expressed in ng/g. Table 4 shows the distribution of cognitive composite scores stratified on the basis of exposure level to Mn and Pb, in the whole cohort and in girls and boys.

Table 5 shows the results of simple and multiple linear regressions between the cognitive composite score and categorized Mn and Pb exposure in cord blood performed on the whole cohort, in boys and in girls. No association was found between the cognitive composite score and the single exposure to Mn (MODEL 1) and Pb (MODEL 2). Also, no relationship between the cognitive composite score and categorized Mn and Pb co-exposure in cord blood was found (MODEL 3). In boys, the effect of the interaction between categorized Mn and Pb co-exposure on cognitive composite score (MODEL 4) was equal to −5.78 (90% CI: −11.17; −0.40). No interaction was found in the whole cohort or in girls. The results of the simple and multiple linear regression between the composite cognitive score and Pb exposure for each level of Mn exposure, in the whole cohort, in boys and in girls, are shown in Table 6. In boys, the adjusted beta coefficient of high Pb exposure when Mn exposure was low was −0.98 (90% CI: −3.79; 1.83) and when Mn exposure was high it was −6.98 (90% CI: −10.93; −3.04). This effect was not confirmed in girls or in the whole cohort.

**Figure 1:**
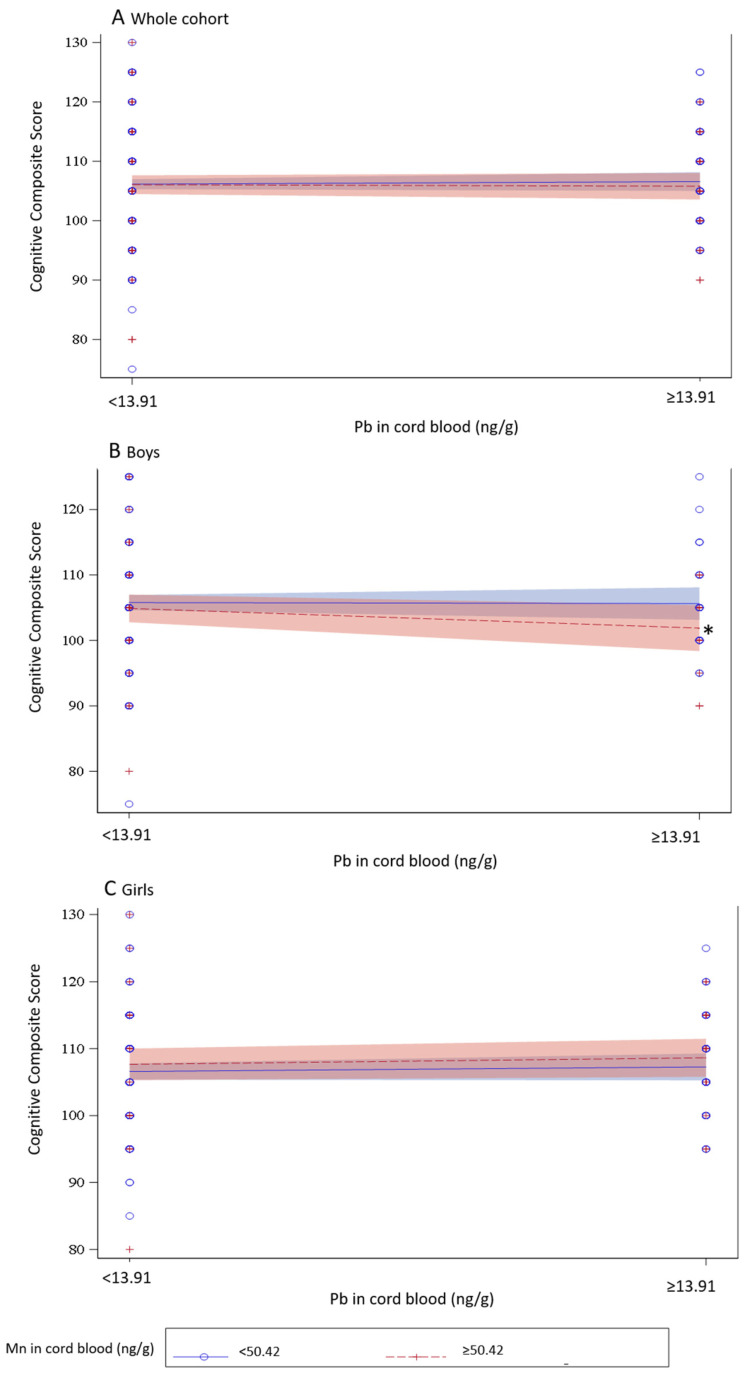
Interaction plot for the effects of Pb and Mn exposure in cord blood on the cognitive composite score, in the whole cohort, in boys, and in girls. * *p*-value < 0.10.

In Figure 1, the interaction plots show an effect on the cognitive composite scores, in the whole cohort (A), in boys (B), and in girls (C) due to the exposure to Mn and Pb. It can be noted that, in boys, the effect of Pb exposure on cognitive composite scores differed by strata (low and high) of Mn exposure.

## 4. Discussion

In this study, we analyzed the relationships between Mn and Pb, and their interaction, and the cognitive neurodevelopment at 18 months of life in an Italian birth cohort that was evaluated previously in relation to other chemical exposures [34,35,38]. The relationship between prenatal exposure to trace elements like Mn and Pb and infant neurodevelopment is complex, and research findings are often inconsistent. As observed in other recent studies [14,15], our results did not suggest any effect of the early prenatal levels of Mn on neuropsychological development. With regard to our results, this may be due to the fact that the observed Mn levels may be within physiological limits, below the threshold point from which this element could cause adverse neurological effects. In fact, for Mn, a non-linear dose–response effect has been suggested by several studies, with a “U-shaped” curve, where both low and high exposures can lead to negative effects, but a middle range is necessary for normal development [39,40,41].

Pb concentrations in cord blood in our cohort (mean ± SD = 12.0 ± 7.4 ng/g, corresponding to 1.13 ± 0.70 µg/dL) were lower than those found in other studies. Smargiassi and colleagues (2002) found a mean of umbilical cord Pb concentrations between 3.2 μg/dL (SD = 2.0) and 1.7 μg/dL (SD = 1.7) [42]. In another recent study, it was reported that, in 200 umbilical cord blood samples, only 12% of them had a Pb concentration below 5.0 μg/dL [43]. Also, we did not find an effect on cognitive neurodevelopment when the co-exposure of Mn and Pb was included in the model. However, when we investigated the interaction effect of Pb and Mn in boys, we found a detrimental effect on cognitive neurodevelopment when both the Pb and Mn cord blood concentrations were high. Conversely, no interaction effect was found when the analysis was restricted to girls. There is increasing evidence in the literature of interaction effects when both the exposures of Pb and Mn are elevated, with a more detrimental outcome than each element alone [44,45]. The differences in the interaction effect observed in the sex-stratified analysis may be attributed to biological, neurodevelopmental, and susceptibility variations between boys and girls, leading to different responses to environmental toxins. Specifically, the absorption and metabolism of trace metals can vary by sex due to hormonal and physiological factors, as well as toxicodynamic differences. Additionally, boys and girls follow distinct brain development pathways, with certain brain regions maturing at different rates based on sex. Moreover, research has shown that children often exhibit sex-specific sensitivities to environmental pollutants, including metals, with hormones like estrogen and testosterone playing a role in both neurodevelopment and the body’s response to toxic exposures [46,47,48,49].

Our findings do not show any association between prenatal exposure to Pb and cognitive neurodevelopment at 18 months, neither when the analyses were conducted in the entire cohort nor when they were performed stratified by sex.

Our study has several limitations. This article focuses on a neurodevelopmental assessment carried out when children were 18 months old: as a result, the full impact of prenatal micronutrient exposure may not yet be completely detectable. Moreover, the connection between trace metals such as Pb and Mn, as well as their co-exposure and interaction effect, and neurodevelopment was only examined at one specific point in time (18 months). Even though several factors were accounted for in adjusting the beta coefficients in our final analysis, neurodevelopment is influenced by numerous factors, some of which remain unknown or unmeasured, such as genetic predisposition [50]. Furthermore, it is possible that some explanatory variables used in the final analysis were inaccurately reported by mothers in the questionnaires—this may lead to an information bias which could introduce residual confounding in the effect estimates.

This study also has several notable strengths. We accounted for multiple covariates and used validated tools to assess the neurocognitive outcome, which were administered by two trained psychologists who demonstrated a high level of agreement. In addition, cord blood trace metal concentrations are ideal sample matrices to assess prenatal and early-life exposure to microelements, and they better represent actual exposure after having crossed the placenta. In our study, we investigated, in the whole cohort and in sex-specific strata, the effect of a single metal, co-exposure to both metals, and their interaction on infant neurodevelopment.

## 5. Conclusions

Overall, these analyses provide a comprehensive understanding of how both individual and combined metal exposures, as well as their potential interactions, contribute to neurodevelopmental outcomes. Our cohort study provides evidence that the interaction of high prenatal exposure to both Pb and Mn is detrimental to early-life cognitive development in boys. However, exact thresholds, safe ranges, and mechanisms remain unclear.

## Figures and Tables

**Table 1:** Main characteristics of 460 mothers in the whole cohort and stratified by the children’s sex.

Main Characteristics of Mothers	Whole Cohort	Boys	Girls
Mother’s age at delivery, mean ± SD (median):	33.2 ± 4.2 (33.0) n = 460	33.1 ± 4.4 (33.0) n = 240	33.3 ± 4.0 (34.0) n = 220
Pre-pregnancy BMI (kg/m^2^), mean ± SD (median):	22.9 ± 4.0 (22.4) n = 460	22.9 ± 3.9 (22.6) n = 240	22.9 ± 4.1 (22.3) n = 220
Maternal IQ mean ± SD (median):	119 ± 11 (125) n = 460	119 ± 12 (124) n = 240	119 ± 11 (125) n = 220
Mother’s marital status at delivery, *n* (%):			
Married/living together	414 (90.8)	214 (89.9)	200 (91.7)
Separated/divorced	13 (2.9)	7 (2.9)	6 (2.8)
Single	29 (6.4)	17 (7.1)	12 (5.5)
Mother’s educational level at delivery, *n* (%):			
Elementary and middle school	86 (18.7)	49 (20.5)	37 (16.8)
High school	219 (47.7)	113 (47.3)	106 (48.2)
University degree	154 (33.6)	77 (32.2)	77 (35.0)
Mother’s occupation at delivery, *n* (%):			
Employed on maternity	353 (77.9)	182 (77.1)	171 (78.8)
Employed worker	33 (7.3)	16 (6.8)	17 (7.8)
Housewife	38 (8.4)	26 (11.0)	12 (5.5)
Other condition	29 (6.4)	12 (5.1)	17 (7.8)
Smoking habits during pregnancy, *n* (%):			
Smoker	40 (8.8)	18 (7.6)	22 (10.1)
Ex smoker	153 (33.8)	78 (33.1)	75 (34.6)
Never smoker	260 (57.4)	140 (59.3)	120 (55.3)
Exposure to passive smoke during pregnancy, *n* (%):			
No	445 (97.0)	233 (97.1)	212 (96.8)
Yes	14 (3.0)	7 (2.9)	7 (3.2)
Surface area of the home of residence, *n* (%):			
<50 m^2^	34 (7.5)	22 (9.3)	12 (5.5)
50–100	308 (67.7)	163 (69.1)	145 (66.2)
>100 m^2^	113 (24.8)	51 (21.6)	62 (28.3)
Area of the home of residence, *n* (%):			
Urban central	166 (36.7)	87 (36.9)	79 (36.6)
Urban suburban	256 (56.6)	135 (57.2)	121 (56.0)
Rural	30 (6.6)	14 (5.9)	16 (7.4)
The home is within 1 km of one or more sources of pollutant missions *, *n* (%):			
Yes	418 (90.9)	218 (90.9)	200 (90.9)
No	42 (9.1)	22 (9.2)	20 (9.1)

* At least one of the following sources of pollutant emissions: industrial activities, highways, state highways, provincial roads, other high-traffic roads, railway station, airport.

**Table 2:** Main characteristics of 460 children in the whole cohort and stratified by the children’s sex.

Main Characteristics of Children	Whole Cohort	Boys	Girls
Birth weight (g), mean ± SD (median):	3419.1 ± 454.2 (3400) n = 459	3480.9 ± 436.6 (3440) n = 240	3351.3 ± 464.2 (3360.0) n = 219
Birth length (cm), mean ± SD (median):	50.1 ± 2.1 (50.0) n = 457	50.5 ± 2.0 (50.0) n = 237	49.7 ± 2.0 (50.0) n = 220
Duration of breastfeeding (months), mean ± SD (median):	10.0 ± 6.0 (10.0) n = 436	10.2 ± 5.9 (11.0) n = 228	9.8 ± 6.2 (10.0) n = 208
AIRE scores:			
Communication and affective interaction between parents and child, mean ± SD (median):	18 ± 2 (19) n = 380	18 ± 2 (18) n = 208	18 ± 2 (19) n = 172
Promotion of child autonomy when alone, mean ± SD (median):	19 ± 2 (19) n = 380	18 ± 2 (19) n = 208	19 ± 1 (19) n = 172
Respect for the child and implementation of rules, mean ± SD (median):	16 ± 2 (17) n = 380	16 ± 2 (17) n = 208	17 ± 2 (17) n = 172
Emotional atmosphere, mean ± SD (median):	17 ± 2 (17) n = 380	17 ± 2 (17) n = 208	17 ± 2 (18) n = 172
Child’s sex, *n* (%):			
Male	240 (52.2)	240 (100.0)	-
Female	220 (47.8)	-	220 (100.0)
Daycare attendance at 18 months, *n* (%):			
Member of the family or other people not included in the family	290 (63.0)	155 (64.6)	135 (61.4)
Kindergarten	170 (37.0)	85 (35.4)	85 (38.6)

**Table 3:** Distribution of cognitive composite scores of 18-month-old children and metal concentrations in cord blood.

	Whole Cohort			Boys			Girls		
	N	Mean ± SD	Median (25th–75th Percentile)	N	Mean ± SD	Median (25th–75th Percentile)	N	Mean ± SD	Median (25th–75th Percentile)
Cognitive composite score	458	106 ± 8	105 (100–110)	240	105 ± 9	105 (100–110)	218	107 ± 8	105 (100–115)
Cord blood (ng/g):									
Mn	460	41.3 ± 15.0	38.5 (30.4–50.4)	240	41.6 ± 14.9	38.6 (30.7–50.4)	220	40.9 ± 15.1	38.5 (29.9–50.3)
Pb	460	12.0 ± 7.4	10.47 (8.1–13.91)	240	11.1 ± 4.6	10.2 (7.7–12.8)	220	13.0 ± 9.4	11.1 (8.4–14.8)

**Table 4:** The distribution of cognitive composite scores of 18-month-old children stratified by dichotomized metal concentrations in cord blood, in the whole cohort and by the children’s sex.

Metals in Cord Blood (ng/g)	Cognitive Composite Score		
	Mean ± SD (Median)		
	Whole Cohort	Boys	Girls
Low exposure to Mn: Mn < 50.42	106 ± 8 (105) n = 344	106 ± 9 (105) n = 180	107 ± 8 (105) n = 164
High exposure to Mn: Mn ≥ 50.42	106 ± 9 (105) n = 114	104 ± 8 (105) n = 60	108 ± 9 (110) n = 54
Low exposure to Pb: Pb < 13.91	106 ± 9 (105) n = 344	106 ± 9 (105) n = 192	107 ± 9 (105) n = 152
High exposure to Pb: Pb ≥ 13.91	106 ± 7 (105) n = 114	104 ± 7 (105) n = 48	108 ± 7 (108) n = 66

**Table 5:** Results of simple and multiple linear regressions between cognitive composite score and Mn and Pb exposure in cord blood.

Metals in Cord Blood (ng/g):	Cognitive Composite Score					
	Whole Cohort		Boys		Girls	
	Crude Beta (90% CI)	Adjusted ^e^ Beta (90% CI)	Crude Beta (90% CI)	Adjusted ^e^ Beta (90% CI)	Crude Beta (90% CI)	Adjusted ^e^ Beta (90% CI)
MODEL 1 ^a^ (*n*):	(n = 458)	(n = 380)	(n = 240)	(n = 208)	(n = 218)	(n = 172)
High exposure to Mn: ≥50.42	−0.27 (−1.76; 1.22)	0.39 (−1.18; 1.96)	−1.67 (−3.76; 0.43)	−1.10 (−3.32; 1.13)	1.29 (−0.80; 3.37)	2.03 (−0.18; 4.25)
MODEL 2 ^b^ (*n*):	(n = 458)	(n = 380)	(n = 240)	(n = 208)	(n = 218)	(n = 172)
High exposure to Pb: ≥13.91	0.20 (−1.29; 1.68)	−0.97 (−2.54; 0.59)	−1.20; −3.47; 1.07)	−2.26 (−4.64; 0.11)	0.92 (−1.05; 2.88)	−0.32 (−2.43; 1.80)
MODEL 3 ^c^ (*n*):	(n = 458)	(n = 380)	(n = 240)	(n = 208)	(n = 218)	(n = 172)
High exposure to Mn: ≥50.42	−0.28 (−2.07; 1.50)	0.47 (−1.41; 2.35)	−1.58 (−3.68; 0.53)	−1.05 (−3.69; 1.60)	1.18 (−0.93; 3.29)	2.11 (−0.56; 4.78)
High exposure to Pb: ≥13.91	0.20 (−1.60; 1.99)	−1.01 (−2.89; 0.86)	−1.04 (−3.31; 1.24)	−2.24 (−5.07; 0.60)	0.77 (−1.21; 2.76)	−0.57 (−3.11; 1.96)
MODEL 4 ^d^ (*n*):	(n = 458)	(n = 380)	(n = 240)	(n = 208)	(n = 218)	(n = 172)
High exposure to Mn: ≥50.42	−0.09 (−1.88; 1.71)	1.04 (−0.84; 2.92)	−0.89 (−3.31; 1.52)	0.20 (−2.29; 2.70)	1.07 (−1.58;3.73)	2.19 (−0.68; 5.07)
High exposure to Pb: ≥13.91	0.44 (−1.35; 2.23)	−0.46 (−2.32; 1.40)	−0.15 (−2.89; 2.59)	−0.70 (−3.47; 2.06)	0.69 (−1.66; 3.04)	−0.51 (−3.04; 2.01)
Mn (high) and Pb (high) interaction	−0.70 (−3.98; 2.57)	−1.92 (−5.39; 1.54)	−2.86 (−7.79; 2.07)	−5.78 (−11.17; −0.40) *	0.29 (−4.09; 4.67)	−0.22 (−4.86; 4.43)

^a^ In MODEL 1, Mn exposure in cord blood was considered. ^b^ In MODEL 2, Pb exposure in cord blood was considered. ^c^ In MODEL 3, both Mn and Pb exposures in cord blood were considered. ^d^ In MODEL 4, Mn and Pb exposures in cord blood and their interaction were considered. ^e^ Adjusted for: maternal IQ, communication, and affective interaction between parents and child, promotion of child autonomy alone, and daycare attendance at 18 months. * *p*-value < 0.10.

**Table 6:** Results of simple and multiple linear regression between Cognitive Composite Score and Pb exposure on each level of Mn exposure.

	Cognitive Composite Score					
	Whole Cohort		Boys		Girls	
Metals in Cord Blood (ng/g):	Crude Beta (90% CI)	Adjusted ^a^ Beta (90% CI)	Crude Beta (90% CI)	Adjusted ^a^ Beta (90% CI)	Crude Beta (90% CI)	Adjusted ^a^ Beta (90% CI)
High exposure of Pb (≥13.91):						
When Mn was low (<50.42)	0.44 (−1.31; 2.19) (n = 344)	−0.45 (−2.31; 1.42) (n = 285)	−0.15 (−2.90; 2.60) (n = 180)	−0.98 (−3.79; 1.83) (n = 158)	0.69 (−1.57; 2.95) (n = 164)	−0.28 (−2.81; 2.24) (n = 127)
When Mn was high (≥50.42)	−0.26 (−3.21; 2.68) (n = 114)	−2.22 (−5.08; 0.65) (n = 95)	−3.01 (−7.12; 1.10) (n = 60)	−6.98 (−10.93; −3.04) * (n = 50)	0.98 (−3.22;5.18) (n = 54)	−0.84 (−5.16; 3.47) (n = 45)

^a^ Adjusted for: maternal IQ, communication and affective interaction between parents and child, tge promotion of child autonomy alone, and daycare attendance at 18 months. * *p*-value < 0.10.

## Data Availability

Due to the database’s complexity and to avoid breaches of the privacy of research participants, the data presented in this study are available upon reasonable request from the corresponding author.
